# Malaria and Cancer: a critical review on the established associations and new perspectives

**DOI:** 10.1186/s13027-021-00370-7

**Published:** 2021-05-13

**Authors:** Toby Ellis, Elvis Eze, Bahijja Tolulope Raimi-Abraham

**Affiliations:** 1 King’s College London, School of Cancer and Pharmaceutical Sciences, Comprehensive Cancer Centre, Guy’s Campus, Great Maze Pond, London, SE1 9RT UK; 2 Malaria no More UK, The Foundry, 17 Oval Way, Vauxhall, London, SE11 5RR UK; 3 King’s College London, School of Cancer and Pharmaceutical Sciences, Institute of Pharmaceutical Science, Waterloo Campus, Franklin Wilkins Building, Stamford Street, London, SE1 9NH UK

**Keywords:** Malaria, Cancer, Comorbidity, Burkitt’s lymphoma, Epstein-Barr virus, Neutropenia

## Abstract

**Objectives:**

Cancer and malaria both have high incidence rates and are leading causes of mortality worldwide, especially in low and middle-income countries with reduced access to the quality healthcare. The objective of this critical review was to summarize key associations and new perspectives between the two diseases as is reported in existing literature.

**Methods:**

A critical review of research articles published between 1st January 2000 – 1st July 2020 which yielded 1753 articles. These articles were screened based on a precise inclusion criteria. Eighty-nine eligible articles were identified and further evaluated.

**Results:**

Many articles reported anti-cancer activities of anti-malarial medicines, including Artemisinin and its derivatives. Other articles investigated the use of chemotherapy in areas burdened by malaria, treatment complications that malaria may cause for cancer patients as well as ways to circumvent cancer related drug resistance. Potential novel targets for cancer treatment, were identified namely oncofoetal chondroitin sulphate and haem, as well as the use of circumsporozoite proteins. A number of articles also discussed Burkitt lymphoma or febrile neutropenia.

**Conclusions:**

Overall, excluding for Burkitt lymphoma, the relationship between cancer and malaria requires further extensive research in order to define association. There great potential promising new novel anti-cancer therapies using anti-malarial drugs.

**Graphical abstract:**

***Created using BioRender*** 
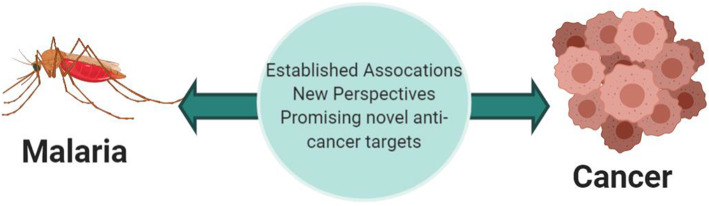

## Highlights

- Excluding Burkitt lymphoma, cancer and malaria are generally described to have an inversely associated relationship.
- With limited research into this relationship being reported in existing literature, any potential association is still up for debate
- There is growing evidence to suggest that some medicines commonly used to treat malaria, including Artemisinin (ART) and its derivatives such as dihydroartemisinin (DHA) and Artesunate (AS), also display anti-cancer properties, implicating these anti-malarial drugs as a possible and promising adjunct to current routine cancer treatment
- Potential novel targets for cancer treatment, were identified namely oncofoetal chondroitin sulphate and haem, as well as the use of circumsporozoite proteins

## Introduction

Comorbidity defined as the co-occurrence of more than one disorder or condition in the same individual [1]. Communicable diseases are otherwise known as infectious or transmissible diseases, whilst non-communicable diseases (NCDs) are mostly chronic diseases such as cardiovascular diseases, cancers, and diabetes; health conditions which are non-transmissible [2]. Strong associations exist between communicable and non-communicable diseases. Addressing the comorbidity associated with NCDs and communicable disease epidemics is important for improved public health outcomes and better economic growth.

Malaria is a communicable disease caused by the Plasmodium family of parasites. In 2018, the World Health Organisation (WHO) reported an estimated 228 million cases of malaria. Malaria infection occurs when a *Plasmodium* infected mosquito bites a human, passing sporozoites (infective spores) into their bloodstream which travel to and invade cells of the liver, where they mature before invading red blood cells [3]. Malaria is caused by parasites of the genus *Plasmodium*, among which five species are known to infect humans: *Plasmodium falciparum*, *Plasmodium malariae*, *Plasmodium ovale*, *Plasmodium vivax* and *Plasmodium knowlesi* [4]*.* Only four known species of *Plasmodium* cause malaria infection in humans; *Plasmodium falciparum,* responsible for the majority of cases in Africa and the Caribbean, *P. vivax*, prevalent (with *P. falciparum)* in Central and South America, Asia, Oceania and the Mediterranean*,* as well as *P. ovale and P. malariae,* both of which are relatively rare [3]*.*

Malaria transmission is rare in developed countries as the anopheles’ mosquito, responsible for malaria infection via the *Plasmodium* species’, tends to reside in warm and arid regions such as those in close proximity to the equator and in LMICs, with hotspots for malaria incidence in Asia and Africa. Notably, above 90% of malaria cases and deaths occur in sub-Saharan Africa, with the most vulnerable age-group being children aged under 5 years for whom around two of three (67%) malaria deaths occur worldwide [4].

Cancer is a condition where cells in a specific part of the body grow and reproduce uncontrollably. Cancerous cells can invade and destroy surrounding healthy tissue, including organs. In 2018 18.1 million cases of cancer, being responsible for around 405,000 and 9.6 million global deaths, respectively [4, 5]. Both cancer and malaria have proven to be leading causes of mortality worldwide. Cancer incidence is relatively equally widespread across the world, though despite being typically lower in LMICs, mortality rates tend to be higher, mainly due to a lack of screening, prevention strategies, widespread vaccination, and treatment. Risk of cancer incidence tends to increase with age, though the incidence of specific cancer types tends to vary by region due to socio-economic factors, cultural awareness and environmental, genetic or lifestyle differences. It is thought that a push towards a more westernized and sedentary lifestyle, which has an increased risk of carcinogenic or radioactive exposure and higher tobacco use, may see a further rise in cancer incidence in LMICs [6].

Despite ongoing improvements to the quality of global healthcare, avoidable deaths are still often attributed to a lack of access to essential treatments, vaccinations, minimal advance in medical technology or a reduced financial capability to afford treatment, especially in low and middle-income countries (LMICs). For example, WHO estimates that approximately 70% of cancer deaths occur in LMICs, with less than 30% of LMICs having the necessary pathology and treatment services required for effective prevention and treatment of cancer, compared to 90% of high-income countries [7].

However, with the aim of providing universal health coverage by 2030, proposed by all United Nations (UN) member states, progress towards nullifying such issues is being made [8]. In terms of malaria, the use of nets treated with insecticides is considered an effective approach in malaria prevention [9]. For cancer, preventing and reducing tobacco smoking to prevent the onset of (tobacco related) cancers, as well as providing cervical cancer screening and human papillomavirus (HPV) vaccinations to aid in earlier diagnosis and treatment for cervical cancer patients have been suggested [8, 10]. A definitive relationship is yet to be established between malaria infection and cancer progression, save for co-infection of malaria with Epstein-Barr virus (EBV) proving to increase one’s risk of developing Burkitt’s lymphoma, a form of non-Hodgkin lymphoma [11].

This critical review aims to investigate the associations between malaria and cancer highlighted in the literature in the last 20 years (between 1st January 2000 – 1st July 2020) in order to establish the influence that infection with malaria may pose to an individual’s risk of cancer development and progression, as well as the impact on cancer treatment and prognosis.

## Methodology

To collect the necessary data required to undertake this literature review, a detailed search was performed using Web of Science, a large database allowing for the ability to access a wide range of scientific literature.

Using Web of Science, the keywords ‘malaria’ and ‘cancer’ were searched for and a citation list was created based on specifically designed inclusion criteria (Table 1). Following this, abstracts of each eligible article were reviewed to exclude literature deemed irrelevant for use in this review.

**Table 1:** The inclusion criteria used to select relevant papers to be included in the literature review

Inclusion Criteria:	Exclusion Criteria:
Keywords “Malaria” AND “Cancer”	Keywords not included
Research articles only	Non research articles (i.e. reviews, book chapter, abstract, PhD/MSc thesis etc. …)
Published between 1st January 2000 – 1st July 2020	Published before 1st January 2000 or on/after 1st July 2020
Written in English	Not written in English
Refined to relate to Malaria within field of oncology	Not relevant in the field of oncology or relating to either malaria or cancer only
Does not meet exclusion criteria	Does not meet inclusion criteria

Following the exclusion of non-eligible publications, each article was reviewed and specific data from each was collected and categorised based on a number of variables; the main research area, whether the methodology used was quantitative or qualitative, the samples used, and populations studied (age, ethnicity, gender or location).

Extracted data could then be used to determine commonly discussed factors mentioned within included articles and in turn, confirm existing associations between malaria and cancer as well as how this may be useful in future research.

## Results

The initial Web of Science search using the keywords “malaria” and “cancer”, prior to applying specifications of the inclusion criteria, produced a total of 1753 search results. The search was then tailored to only display papers between 1st January 2000 – 1st July 2020, producing 1661 results. Thereafter, the search was refined to display “articles” only, producing 1129 articles, and then condensed further to ensure all articles were written in “English”, producing 1117 articles. Finally, given the vast number of search results and to further ensure specificity, the search was refined to only include articles in the research area of “oncology”, providing a total of 119 articles.

The results of the Web of Science search, upon applying the inclusion criteria, have been simplified and displayed in Table 2.

**Table 2:** Search results upon applying specific search criteria to refine the Web of Science search results to fit the specifically designed inclusion criteria (in Table 1)

Search Criteria:	No. Search Results:
Keywords: “Malaria” and “Cancer”	1753
Publication Years “2000–2020”	1661
Document Types: “Articles”	1129
Languages: “English”	1117
Research Areas: “Oncology”	119

To further guarantee specificity to the relationship between malaria and cancer, the abstract of each paper was reviewed, with a total of 30 articles being deemed irrelevant and excluded. Consequently, a final total of 89 articles were selected to be included in this structural review.

Once selected, relevant data from each article was extracted. Using this data, areas of critical importance pertaining to the relationship between malaria and cancer were highlighted and investigated further.

**Fig. 1:**
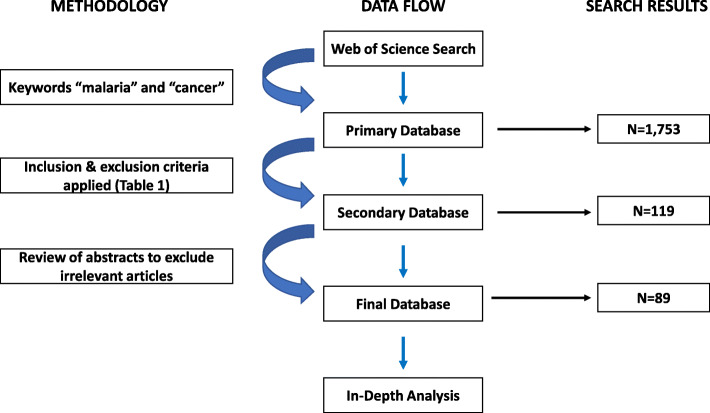
Simplified schematic representation of the systematic review process used in this literature review

The systematic review process and initial results have been simplified and presented in Fig. 1.

### Populations studied in included articles

**Fig. 2:**
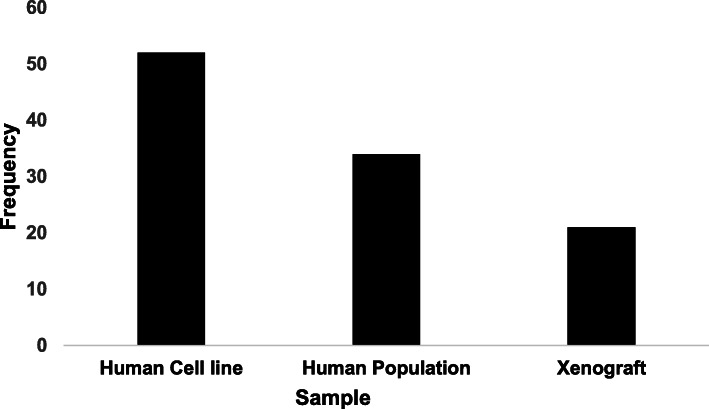
Frequency of sample type used in included articles

**Fig. 3:**
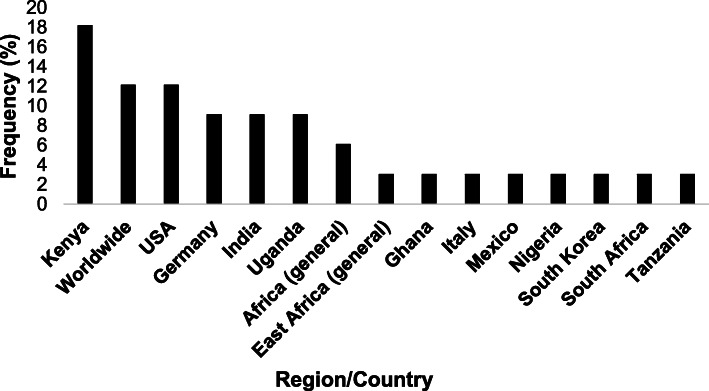
Frequency (by percentage) of each Region/Country studied in included articles that sampled human populations

Of the 89 research articles reviewed, 58.4% (*n* = 52) used human cell lines in their analysis. 23.5% (*n* = 21) used xenograft models and of these studies 90.5% (*n* = 19) used mouse models and the remaining two studies used a rabbit model and a zebrafish model, respectively. Eighteen studies used a mixture of in vivo and in vitro samples, included in the total count for both calculations (Fig. 2). The remaining studies investigated human populations (*n* = 34) with studies being conducted across a number of continents (*n* = 4; Africa, Asia, Europe and North America) and in a range of countries (*n* = 14; listed in Fig. 3).

Notably, half (*n* = 18) of the articles using human populations, whether healthy or diseased individuals, were conducted in Africa countries, specifically studying populations in East African countries, including Kenya (*n* = 6), Uganda (*n* = 3) and Tanzania (*n* = 1), West African countries, including Nigeria (*n* = 1) and Ghana (1), and in South Africa (1). Moreover, three articles focussed on the entire African continent and one on the region of East Africa entirely. No articles included in this review sampled populations in Northern or Central Africa.

Several studies were also conducted in Asian countries (*n* = 4), namely India (*n* = 3) and South Korea (*n* = 1), where malaria incidence still remains prevalent. Several studies were conducted in Europe (*n* = 4), specifically in Germany (*n* = 3) and Italy (*n* = 1), and North America (*n* = 5), specifically in the USA (*n* = 4) and Mexico (*n* = 1), where malaria incidence is low. Notably, despite malaria remaining an issue in a number of South American countries, no articles included in this review studied South American populations, possibly due to the majority of South American countries being Spanish or Portuguese speaking as opposed to English (Fig. 3).

Of the articles studying human populations (*n* = 35), almost half (42.9%) used child participants under the age of 18 (*n* = 15) and a third used adult participants (*n* = 12). The remainder of studies either included both adults and children (*n* = 5) or did not specify the ages of sampled individuals (*n* = 3).

### Research areas discussed in included articles

The majority of included articles discussed the use of anti-malarial medicines for treating cancer (*n* = 51), highlighting the importance of research in this area when discussing the associations between cancer and malaria. Of these articles, the majority focussed on Artemisinin and its derivatives (*n* = 35), including artesunate and dihydroartemisinin, while a small number (*n* = 4) displayed the use of anti-malarial medicines used in traditional Asian healthcare, namely Rhus javanica Linn, Pulsatilla koreana, *Alstonia scholaris* Linn R Br and Harmol, customarily used in eastern Asian countries, including China and South Korea.

Multiple articles investigated other forms of treatment (*n* = 12), for example, the effectiveness of chemotherapy in malaria-burdened areas (*n* = 1), as well as complications that malaria may cause for cancer patients (*n* = 1) and methods of circumventing cancer related drug resistance (*n* = 3). Furthermore, a number of articles proposed novel targets for cancer treatment, including the glycosaminoglycan, oncofoetal chondroitin sulphate (*n* = 2), and haem (*n* = 1), as well as two articles targeting NF-κB for inhibition using circumsporozoite proteins typically found in *Plasmodium* species (*n* = 2). Notably the use of sporozoites in cancer treatment was further investigated in two separate studies (*n* = 2), one demonstrating the antigen specific CD8+ T cell response induced upon infection and the other indicating its prodrug mediated cell killing ability.

**Fig. 4:**
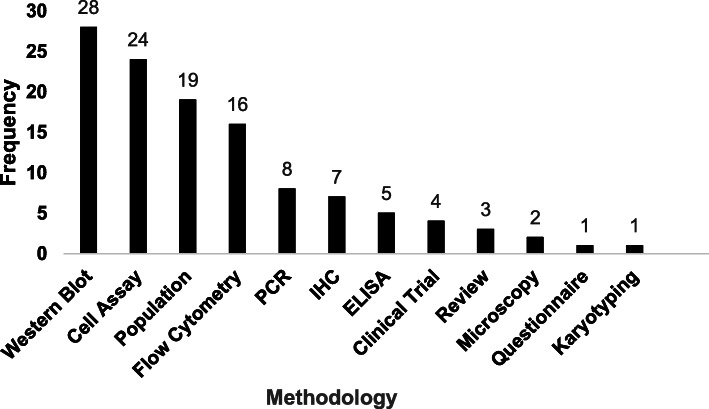
Frequency of methodologies used in eligible articles

Overall, the vast majority of included articles focussed on treating cancer (*n* = 63), while the remainder (*n* = 27) described associations between cancer and malaria, with a number of these articles investigating Burkitt lymphoma (*n* = 14) and the remainder discussing either the general relationship between malaria with cancer (*n* = 12) or febrile neutropenia (*n* = 3), a low neutrophil count with a fever (Fig. 4).

### Methodologies used in included articles

The majority of included articles (*n* = 82) used quantitative methods, while the remainder (*n* = 7) used qualitative methods. None of the studies included in this review used a mixture of both (specific methods detailed in Fig. 4). Of the quantitative approaches, a multitude of procedures were mentioned including performing a cell assay (*n* = 24) recording features such as cell proliferation, apoptosis, migration and invasion, as well as performing a western blot (*n* = 28), flow cytometry (*n* = 16), PCR (*n* = 8), IHC (*n* = 7) or ELISA (*n* = 5), with a number of studies using a mixture of methods.

Many of the population-based studies analysed epidemiological differences (*n* = 19), for example comparing cancer incidence in areas with a high malaria burden against those with no or low malaria transmission. Moreover, the efficacy and safety of using artesunate for the treatment of cancer patients was assessed in a small number of clinical trials (*n* = 4); administered orally for breast cancers (*n* = 2), intravaginally for CIN (*n* = 1) or intravenously for solid tumours (*n* = 1).

In contrast, articles that opted for qualitative research methods utilised meta-analyses (*n* = 3), microscopic observations of *Plasmodium falciparum* (*n* = 2), a questionnaire (*n* = 1) regarding the history of both malaria and cancer diagnosis and karyotyping for genetic mutations (*n* = 1).

## Discussion

The areas of key importance most frequently discussed among included articles consist of; the burden of endemic Burkitt lymphoma in sub-Saharan Africa, associations between malaria and cancer generally, the use of anti-malarial medicines in cancer treatment and other methods related to cancer treatment.

### Burkitt lymphoma

Burkitt lymphoma (BL) was first described by Denis Burkitt in the 1950’s. While working in Uganda, he observed numerous children with large jaw tumours (usually observed in endemic BL), subsequently noting that the geographical distribution of these tumours tended to correspond to that of malaria. BL has been strongly associated with Epstein-Barr virus (EBV) infection, however, given that EBV is found ubiquitously, EBV infection alone does not explain the burden of BL in Africa but rather, is hypothesised to act as a co-factor for BL along with malaria infection [12].

BL is a B-cell malignancy common among African children, with incidence rates in sub-Saharan Africa being especially high (endemic BL) due to countries closer to the equator having warm, tropical climates suitable for the anopheles’ mosquito. In countries outside of the equatorial belt, incidence tends to be lower (sporadic BL), similar to those in high-income countries. BL can also be caused by HIV infection, although whilst endemic BL tends to only affect children, sporadic and HIV-associated BL can develop at any age and are much less associated with EBV infection than endemic BL (responsible for around 30% of sporadic BL cases and rarely HIV-associated BL) [13].

A number of articles focussed on BL incidence and any contributing factors, co-factors or survival factors deemed influential to its onset including geography, ethnicity, age, sex and more specific factors such as nutrition, anaemia, malaria endemicity and EBV viral load.

EBV is reported to be a co-factor for BL alongside malaria. Firstly, one study analysing this relationship recorded location-specific rates of EBV, correlating high and low risk clusters with BL incidence rates. It was deduced that for rates of EBV infection and BL incidence, spatial clustering patterns were positively correlated. This discovery, alongside malaria transmission and BL incidence being positively correlated, further confirms the suggestion that endemic BL can be caused by concurrent malaria and EBV infection [14]. Secondly, in a study using mice displaying immunity against malaria, following continuous antigenic stimulation by *Plasmodium yoelii-yoelii,* subjects were shown to develop neoplasms histologically similar to BL observed in humans*,* further confirming consistent malarial and viral infection as contributing to BL pathogenesis [15]. The study concluded that the immune response of a host against malaria parasites can be viewed as the start of Burkitt’s lymphoma with the parasites and viral agents acting as inducers of the host response [15]. EBV as a risk factor in malaria was further investigated in two studies comparing serological profiles of healthy individuals and BL patients to determine differences in anti-EBV antibody responses. Notably, both studies determined anti-EBV antibody concentrations were increased for individuals with BL, indicating that EBV infection was more frequent than in healthy controls and thus, may indeed play a role in BL pathogenesis [16, 17]. In addition, findings from both studies suggest that EBV and malaria may act in a synergistic way in the pathogenesis of childhood Burkitt lymphoma [16, 17].

One Kenyan study found that for children diagnosed with BL, elevated serum lactase dehydrogenase (LDH) and decreased EBV viral load was associated with increased risk of relapse or death as well as for children with anaemia, who are malnourished or have a high grade tumour. Despite acknowledging EBV as a co-factor for endemic BL, this study found no association between baseline EBV load and mortality [18]. However, in a similar study, elevated EBV viral load and an increased malaria burden were observed in high risk regions of Kenya for endemic BL compared to lower risk regions, further adding to the evidence attributing endemic BL pathogenesis to EBV and malaria co-infection. Chronic malnutrition was again confirmed as a BL risk factor in children, with low levels of glutathione peroxidase (GPx), a biomarker representing nutritional status, indicating malnourishment [19]. Children from endemic BL high incidence regions appeared to have significantly low GPx levels and high EBV viral load [19]. This trend was also observed in children with *P.falciparum* infection [19]. The study also suggested an associated selenium deficiency as an additional risk factor for BL [19]. In addition, two studies investigating BL incidence rates, one in Tanzania and one in the US (assessing sporadic BL), deduced that male sex, young age (peaking highest between 3 and 5 and second between 6 and 9 in the US and 5–9 in Tanzania) and early exposure to malaria and EBV were found to be significant risk factors for BL [20, 21].

Furthermore, in a study assessing genetic profiles of BL patients (rather than serological profiles), decreased levels of proteins related to the immunoproteasome complex and altered PTEN/PI3K/mTOR signalling was associated with EBV type 1 mutations, with multiple genes found to commonly be mutated (TFAP4, MSH6, PRRC2C, BCL7A, FOXO1, PLCG2, PRKDC, RAD50, and RPRD2*).* Increased mutations, reduced protein degradation and reduced control of cell proliferation in patients with EBV type 1 mutations (as opposed to those with no mutations) supports the role of EBV (type 1) infection in BL oncogenesis [22]. Furthermore, in a study comparing T-cell responses in healthy children and endemic BL patients, overall Epstein-Barr nuclear antigen 1 (EBNA-1) specific responses were significantly reduced in BL patients, indicating that loss of specific immunosurveillance for EBV may occur in patients who develop BL and thus, are unable to produce a sufficient immune response upon EBV infection [23].

Specifically, *P. falciparum* has been linked to BL pathogenesis via a variety of mechanisms. For example, *P. falciparum* parasites absorb vitamin A from the host cell, with alterations to vitamin A metabolism being implicated in a number of cancers including BL. Retinoic acid, a vitamin A metabolite, is involved in B cell proliferation, maturation and regulation and in turn, the expression of activation-induced cytidine deaminase (AID) by B cells. Upon persistent *P. falciparum* infection*,* an individual may be exposed to toxic levels of retinoic acid causing increased B cell expansion and in turn, increased AID expression which itself increases the risk of c-myc translocations, a common mutation in BL [24]. It is important to note that this association would not be dependent on geographical location if genetic variability is accounted for.

Individuals carrying the sickle-cell trait have previously been shown to confer resistance to malaria. One study comparing BL incidence between populations with the sickle-cell trait (HbAS) and healthy controls (HbAA) reported significantly increased incidence rates in children in areas where malaria is holoendemic regardless of sickle-cell trait prevalence, suggesting it provides no protection against BL itself [25].

### The general association between malaria and Cancer

Excluding Burkitt lymphoma, cancer and malaria are generally described to have an inversely associated relationship. However, with limited research into this relationship being reported in existing literature, any potential association is still up for debate [26].

This inverse association is displayed in a longitudinal study correlating malaria incidence and all-cause, age-standardised cancer mortality worldwide (56 countries over 6 continents; 20 European, 11 South American, 10 Asian, 7 North American, 6 African and 2 Oceanic) for 30 types of cancer over 53 years. Through comparing WHO statistics for malaria incidence and cancer mortality and applying a generalised additive mixed model (GAMM), displaying the relationship between the two variables while accounting for non-Gaussian distribution as well as for factors including; ethnicity, economic and healthcare development and life expectancy. Endemic or epidemic malaria was shown to significantly decrease mortality for colon cancer in both men and women, as well as lung cancer and stomach cancer in men and breast cancer in women [26].

Furthermore, one study displayed that in mice with Lewis cell lung cancer, exosomes specifically produced in Plasmodium-infected hosts suppressed tumour angiogenesis with evidence of reduced expression of vascular endothelial growth factors (VEGF), a novel discovery displaying the inverse association between malaria and cancer [27]. Moreover, tumour necrosis factor polymorphisms have been associated with severe malaria in African countries, most notably for this review, TNF-238 polymorphisms. Two separate studies investigated how TNF-238 polymorphisms affected cancer susceptibility, one specifically focussing on Hodgkin lymphoma and the other covering a range of cancers (gastric, cervical, colorectal and renal). TNF-238 polymorphisms were reported to play a protective role against cervical cancer and are associated with a reduced risk of developing Hodgkin lymphoma as well as cervical, gastric, renal and colorectal cancers, again suggesting an inverse association [28, 29].

However, this proposed association was disputed in a study comparing malaria incidence, using statistics provided by the CDC, and all-cause cancer mortality, using NCI statistics, in the US between 1950 and 1994. This study found a significant association between malaria incidence and cancer mortality independent of varying population size, median age and percentage of African Americans (considered due to higher prevalence of sickle cell trait) residing in each state. This association was instead attributed to the ability of Plasmodium to suppress the immune system and thus, increase the risk of developing a secondary infection or disease, in this case, cancer [30].

Notably, one study described a specific association between malaria incidence and risk of non-Hodgkin lymphomas such as Burkitt Lymphoma, although not with Hodgkin lymphomas, based on a case-control study of lymphoma patients compared to healthy individuals. Increased risk of non-Hodgkin lymphomas was attributed to previously suffering from infectious mononucleosis, which itself supports the theory of EBV and malaria co-infection in Burkitt lymphoma pathogenesis [31].

In two population-based studies investigating malaria as a risk factor for cervical cancer in Africa, one in Nigeria and one in Uganda, conflicting results were discovered. In Uganda, cervical cancer incidence rates were significantly higher in regions with higher malarial endemicity, with a higher percentage of high grade cancer [32], while in Nigeria, no significant association was described between self-reported malaria and cervical cancer, although as incidence was recorded via questionnaire, cases may have gone undiagnosed or unreported [33]. A lack of consistency within results demonstrates that further research in this field is required.

Febrile neutropenia (FN) is described as symptoms of a fever in a patient with an abnormally low neutrophil count and is a predominant issue and cause of mortality, especially for patients treated via chemotherapy. FN is commonly caused by parasitic infection, however, in three separate studies investigating whether malaria infection is a causative factor for FN in leukaemia and lymphoma patients undergoing chemotherapy, results were mixed. One study in India concluded that although malaria is not a causative agent for FN, a small number of patients with FN episodes had previously suffered from malaria [34, 35]. In contrast, the remaining two studies maintained that malaria is indeed a complicating factor of FN for patients undergoing chemotherapy and could be a causative factor [34, 36]. This lack of consistency again highlights the need for further research in this field.

Notably, one study investigated Duffy antigen receptor for chemokines (DARC) expression for NSCLC patients using a human adenocarcinoma cell line (A549). DARC has previously been identified as a coreceptor for malaria, highlighting that overexpression of DARC increases one’s susceptibility to malaria infection. Tumours expressing DARC were found to be larger in size and have significantly more necrosis present than control tumours. However, DARC-expressing tumours were also shown to display decreased tumour cellularity and decreased tumour-associated vasculature and, in turn, a reduced ability to metastasise. These results indicate that DARC-expressing tumours, as well as increasing an individual’s susceptibility to malaria infection, may grow more aggressively than non-DARC-expressing NSCLC tumours but may also be less likely to metastasise [37].

### Anti-malarial medicines in Cancer treatment

There is growing evidence to suggest that some medicines commonly used to treat malaria, including Artemisinin (ART) and its derivatives such as dihydroartemisinin (DHA) and Artesunate (AS), also display anti-cancer properties, implicating these anti-malarial drugs as a possible and promising adjunct to current routine cancer treatment.

Artemisinin is an active extract taken from the wormwood plant (*Artemisia annua* L.) and since its initial use in traditional Chinese medicine for treating fevers, it has been synthesised and implemented into routine malaria treatment and since, has also been shown to suppress cancer cell growth, most commonly in leukaemia and lymphomas. Notably, multiple analogues of ART have since been developed with improved pharmacological properties, the most prominent being Artesunate (AS), currently recommended by WHO for the treatment of severe malaria. In patients with leukaemia, Artemisinin’s have been shown to induce cell cycle arrest, activate apoptosis via ROS-dependent mechanisms and disrupt lysosomal behaviour, mechanisms which have been further reported in both xenograft models, with reduced overall tumour growth, and AML cell lines, with reduced cell proliferation and increased apoptosis [38–40].

Further studies reported the value of ART in cancer treatment, with one study reporting increased cell cycle arrest and apoptosis and reduced cell growth and proliferation against hepatocarcinoma (Hep-G2) and cholangiocarcinoma (CL-6) cell lines, notably with increased sensitivity than fluorouracil (5-FU), a standard anti-cancer drug [41]. Similarly, a study investigating using ART in treating neuroblastoma reported significantly inhibited cell growth and proliferation of three separate human neuroblastoma cell lines (SHEP1, SK-N-AS and SK-N-DZ), with increased cancer cell apoptosis and reduced tumour angiogenesis and migration, further displaying the ability for ART to reduce both tumour growth and the risk of metastasis [42].

AS, a semi-synthetic derivative of ART, is also reported to display potent anti-cancer activity, most commonly in preventing leukaemic cell growth and inducing apoptosis, possibly through inducing a DNA damage response or preventing homologous recombination, ultimately resulting in double-strand DNA breaks and in turn, tumour cell death. AS has also displayed prominent anti-lymphoma activity against a wide range of B-cell lymphoma cell lines [43–47].

Treatment using AS may also be useful in circumventing multi-drug resistance, being active against typically chemoresistant cancers (including renal tumours and cancers of the CNS) and especially active against drug-resistant cell lines for both leukaemia and colon cancers. Notably, one study found that treatment with AS had the ability to reverse multi-drug resistance (proved with a cell viability assay) in an oesophageal cell line (Eca109/ABVG2) typically resistant to chemotherapy. The cytotoxic activity of AS against a range of cancers may prove it to be a useful adjunct to chemotherapy, especially in typically chemoresistant cancers [48–50].

The value of AS in cancer treatment was further confirmed in a number of clinical trials, with four articles reporting the results of phase I trials, the purpose of which were to calculate the maximum tolerated dose (MTD) and dose-limiting toxicities (DLT) of treatment using AS following proof of anti-cancer activity in a range of cancers (including the NCI-60 cell lines, a panel of 60 different human cell lines used by the National Cancer Institute). Two trials investigated using AS in breast cancer, one in cervical cancer (CIN 2/3) and one investigating solid tumours generally. In all trials, AS displayed prominent anti-cancer activity while proving the safety of AS administration, orally, intravaginally and intravenously, with relatively few DLTs at planned increased doses (15% for BC, 20% for solid tumours) that also tended not to interrupt the trial or require intervention. Issues only arose for treating CIN 2/3, where 90% of patients experienced adverse effects, although only 1 patient (2.5%) experienced a severe DLT. For the general treatment of solid malignancies, intravenous administration of AS was limited to a maximum dose of 18 mg/kg, where for both breast cancer and CIN, a maximum oral dose of 200 mg daily was recommended [51–54].

The anti-cancer properties of AS have also been investigated in a number of studies using a mixture of in vivo and in vitro methods in order to determine the specific mechanisms behind this activity. AS has been shown to reduce cell proliferation and induce apoptosis in multiple cancers via a number of different mechanisms; Merkel cell carcinomas (via ferroptosis, a new type of cell death which is iron dependent and is accompanied by large amounts of iron accumulation and lipid peroxidation during cell death) [55].

oesophageal cancers (via Bcl-2 downregulation and Bax and caspase-3 upregulation), glioblastomas (determined via cell viability assay) and gastric cancers (via COX-2 downregulation). The fAS has also been shown to play a protective role against cerebral ischemia-reperfusion injury by inhibiting oxidative and inflammatory processes that can cause neuronal damage, and exhibits specific toxicity in a dose-dependent manner against retinoblastoma cells (inhibiting iron uptake by CD71) [56–61].

Similar to ART and AS, the anti-cancer activity of DHA has been studied in a range of human cancer cell lines. DHA was shown reduce cell proliferation, induce apoptosis and suppress cancer cell migration and invasion in bladder cancer (cell cycle arrest via p21 and KDM3A regulation) and ovarian cancer (inhibits abnormal hedgehog pathway activation) as well as specifically increasing apoptosis in colon cancer (activates Janus Kinase 2 induce apoptosis via MAPK), prostate cancer (by upregulating the TNF death receptor 5) and endothelial cell cancers (activates JNK/SAPK pathway to express pro-apoptotic factors) [62–66].

DHA may also be useful as an adjunctive therapy alongside chemotherapy, having overcome chemoresistance in multiple myeloma (to dexamethasone) and Lewis lung cell carcinoma, while also generally inducing cell cycle arrest via p-38 MAPK activation, increasing sensitivity to carboplatin therapy [67, 68]. Moreover, DHA may be promising in treating aggressive breast cancers, having been shown to act synergistically with both Doxorubicin, for triple-negative breast cancers, and Trastuzumab, for HER2+ breast cancers, while displaying the ability to bind to translationally controlled tumour proteins (TCTP) which tend to be overexpressed in high grade breast cancers and tumours conferring resistance to treatment using Trastuzumab. This again highlights the ability of DHA to circumvent drug resistance [69].

Notably, given the high incidence and poor prognosis of NSCLC worldwide, the ability for ART and its derivatives to inhibit the Wnt/β-catenin pathway may be vital to improving mortality rates. Inhibition of the Wnt/β-catenin pathway by ART prevents NSCLC tumorigenesis (via Cyclin D1 production) and epithelial-mesenchymal transition (via E-cadherin production) and also reduces cell migration (via matrix metalloproteinase production), again displaying the value of using ART to reduce tumour proliferation and metastasis [70]. Similar to ART, AS was also shown to inhibit the Wnt/β-catenin pathway but in a separate study on uveal melanomas and was also confirmed in another study to be a useful adjunct for two uveal melanoma patients [71, 72].

Notably, one other derivative of ART, Artemisone, displayed prominent anti-cancer activity in vitro for a number of cancer cell lines and, similar to ART and its other derivatives, may be a useful adjunctive cancer therapy [73].

The anti-malarial drug, Quinacrine, has also been reported to have anti-cancer activity, especially against cancers in which p53 is mutated, due to its ability to activate p53 signalling pathways and resume normal tumour suppression, as well as suppressing NF-κB and inhibiting topoisomerase activity [74, 75].

Moreover, in a number of studies assessing the promise of other anti-malarial drugs (including Chloroquine, Mefloquine and Pyrimethamine) in cancer treatment, results displayed that anti-malarial drugs generally tended to reduce cell proliferation and migration while increasing apoptosis in a range of cancers, overall reducing the risk of tumour formation and development through a number of mechanisms [76–82]. As well as suppressing overall tumorigenesis, anti-malarial drugs (including ART derivatives) were reported to be useful in circumventing chemoresistance with possible mechanisms suggested to include the inhibition of enzymes such as glutathione S-transferases or mediation via proteins such as P-glycoproteins [83, 84].

Surprisingly, two studies implicated treatment using anti-malarial drugs in increasing tumorigenesis, one suggesting that Pyrimethamine can cause chromosomal alterations, with the risk of mutation increasing in a dose-dependent manner [85]. The other study deduced that treatment using Pyrimethamine, as well as using Chloroquine or Primaquine, may stimulate cell proliferation and may therefore have a tumour-promoting effect [86].

Four articles studying the use of anti-malarial medicines conventionally used in traditional Eastern Asian medicines (previously listed) reported cytotoxicity towards cancer cells, mainly through inducing apoptosis. Notably, three of the medicines studied (excluding ASLRB) interacted with caspases in its anti-cancer mechanisms, with RJL and PK extract regulating caspase-3 expression among other pro-apoptotic genes while Harmol activated caspase-8 [87–90].

### Other methods of Cancer treatment discussed

There is limited research discussing the various complications malaria may cause for a patient receiving treatment for cancer and, aside from the use of anti-malarial agents, how existing knowledge of malaria could be utilised to further aid and improve the quality of cancer treatment.

One study in India analysed 30 patients receiving treatment for solid tumours and deduced that malaria posed a significant issue for these patients, leading to delays in routine treatment and a number of complications (33 overall), including, but not limited to, thrombocytopenia, anaemia, dysfunction of the kidneys and reduced blood pressure, which while none proved to be fatal, posed a considerable threat to the patient’s health and the efficiency of treatment [91]. This risk for cancer patients is further displayed in a study in Malawi on 20 patients with Wilms tumours, a common childhood kidney cancer, in which one patient died as a result of malaria infection [92].

However, previous studies have also reported on the ability of the Plasmodium circumsporozoite protein (CSP), present on the surface of malaria parasite sporozoites, to inhibit NF-κB, a transcription factor commonly implicated in inflammation and cell proliferation and survival. In two separate studies, the CSP suppressed cell growth in lung cancer (A549 cells) and colorectal cancer (SW480 cells) via NF-κB inhibition [93, 94]. Furthermore, one promising anti-cancer vaccine mechanism involves provoking an anti-tumour immune response using malaria parasite sporozoites via knocking down specific genes, namely genes upregulated in infective sporozoites (UIS3/4) or involved in fatty acid synthesis (Fabbf). This method has been shown to attenuate the sporozoite in lung cancers, thereby inducing an antigen-specific CD8+ T cell response that suppresses tumour growth [95, 96].

Oncofoetal Chondroitin Sulphate (oCS) is a receptor for VAR2CSA, a protein implicated in placental binding of malarial infected erythrocytes that allows for the parasite to exit circulation, and has itself been reported to be expressed in the majority of cancers. Three studies highlighted (oCS) as a target for anti-cancer therapy, suggesting that oCS modification may be linked to cancer cell motility. One study administered a drug conjugate of VAR2CSA that successfully killed Burkitt lymphoma cells in vivo while the other two used a recombinant VAR2CSA-protein to inhibit tumour activity, all demonstrating the promise of oCS as a target for novel cancer therapies [97–99]. Furthermore, one study displayed the promise of a recombinant fusion protein consisting of a malaria circumsporozoite protein for receptor mediated drug delivery [100].

Haem has also been suggested as a promising target for anti-cancer therapy and is expressed in a multitude of different tumour types and notably, serves as the target for the anti-malarial ART which itself has been reported to display anti-cancer activity. One study identified a number of haem-interacting compounds that are cytotoxic towards human leukaemia cell lines, indicating that haem may be a useful target for a novel anti-leukaemic therapy [101].

## Conclusion

Cancer and malaria are responsible for high incidence and mortality rates worldwide, especially in low and middle-income countries with reduced access to the quality of healthcare required to deal with such burdens.

Through reviewing 89 articles published in oncological journals between 1st January 2000 – 1st July 2020, this literature review aimed to summarize associations commonly reported in order to gain a greater overall understanding of the relationship between malaria and cancer.

The onset of endemic Burkitt lymphoma (as well as in around 30% of sporadic BL cases) is by far the most evident association between cancer and malaria, with co-infection by EBV and malaria being responsible for BL pathogenesis via a number of proposed mechanisms including vitamin A depletion, genetic mutations and immunosuppression.

When discussing this relationship generally however (excluding BL), results were varied, with malaria tending to cause complications (including febrile neutropenia) for patients receiving cancer treatment who are immunosuppressed although in a number of studies, mechanisms of tumorigenesis tended to be suppressed, including angiogenesis, cell proliferation, invasion and migration.

Interestingly, Artemisinin and its derivatives, including artesunate and dihydroartemisinin, drugs typically used for treating malaria, display prominent anticancer activity against a range of human cancer cell lines as well as in xenograft models (i.e. mice), including reducing cell migration, invasion and proliferation and inducing apoptosis. A number of anti-malarial medicines used in traditional Asian healthcare also displayed similar cytotoxicity towards cancer cells. Furthermore, Artemisinin’s and a number of other anti-malarial drugs showed a promising ability to circumvent cancer drug resistance and thus, may be a useful adjunct to chemotherapy.

Moreover, evidence suggests that the Plasmodium circumsporozoite protein (CSP) can inhibit NF-κB and thus, suppresses cell survival and proliferation and furthermore, that knocking down specific genes in sporozoites may induce an antigen-specific CD8+ T cell response that suppresses tumour growth. Several studies also suggested promising targets for anti-cancer therapies; oncofoetal chondroitin sulphate, a receptor for VAR2CSA, and haem, the target for Artemisinin which itself expressed in a number of cancer types.

Overall, excluding Burkitt lymphoma, associations between cancer and malaria are yet to be extensively reported, though it is suggested that malaria infection can cause complications for patients already undergoing cancer treatment, however, may potentially suppress tumorigenesis in otherwise healthy individuals. To confirm this general relationship more clearly, global epidemiological studies comparing malaria and cancer incidence for a wide range of cancer types should be performed, with evidence from in vivo and in vitro studies aiding to determine the specific mechanisms behind any trends observed.

There is potential for novel anti-cancer therapies in the field of malaria parasitology including potential cancer treatment targets as well as evidence of anti-malarial medicines being cytotoxic towards several human cancer cell lines and effective in a small number of clinical studies. Potential treatment targets should be further explored for a potential basic discovery to aid in chemotherapy delivery while use of anti-malarial drugs for cancer therapy should be further investigated in clinical trials, xenograft models and human cancer cells lines to confirm their safety and efficacy as well as mechanisms of action as an adjunct to chemotherapy. The global health significance of finding common ground for a communicable disease like malaria and cancer - which is generally classified as non-communicable - may bring new perspectives to care delivery within adaptable health systems. Importantly, in LMICs, the potential for discovering low-cost therapeutics for cancer may provide an opportunity for broad-based quality improvements.

## Data Availability

The data used in this study are available from the corresponding author upon request.
